# Evaluation of cottonseed oil as a substitute for fish oil in the commercial diet for juvenile swimming crabs (*Portunus trituberculatus*)

**DOI:** 10.1016/j.aninu.2024.07.004

**Published:** 2024-08-29

**Authors:** Tiantian Xu, Zheng Yang, Shichao Xie, Tingting Zhu, Wenli Zhao, Min Jin, Qicun Zhou

**Affiliations:** Laboratory of Fish and Shellfish Nutrition, School of Marine Sciences, Ningbo University, Ningbo 315211, China

**Keywords:** Swimming crab, Cottonseed oil, Lipid metabolism, Volatile substances

## Abstract

A six-week feeding trial was carried out to determine the feasibility of cottonseed oil (CSO) as a viable substitute for fish oil (FO) in the commercial diet of swimming crabs. Ninety healthy swimming crabs (initial body weight 34.28 ± 0.59 g) were randomly assigned to 90 plastic baskets. Three isonitrogenous and isolipidic diets (450 g/kg protein and 120 g/kg lipid) were formulated replacing FO with CSO at 0%, 50% and 100% (CSO-0, CSO-50, and CSO-100), respectively. Each diet was randomly allocated to three replicates, each consisting of 10 crabs. Results indicated that crabs fed with CSO-100 diet had the lowest the percent weight gain (PWG), specific growth rate (SGR) and survival among all treatments (*P* < 0.05). Albumin (ALB), glucose (GLU), triglyceride (TAG), total cholesterol (T-CHO), low-density lipoprotein cholesterol (LDL-C), non-esterified fatty acid (NEFA) contents and alkaline phosphatase (ALP), alanine amino transferase (ALT) activity in hemolymph were significantly affected by dietary substitution of FO with CSO (*P* < 0.05). The contents of total saturated fatty acids (SFA), total mono-unsaturated fatty acids (MUFA) and total long-chain polyunsaturated fatty acids (LC-PUFA) in the hepatopancreas and muscle were negatively correlated with the substitution level, whereas total n-6 polyunsaturated fatty acids (n-6 PUFA) and linoleic acid (18:2n-6) contents increased significantly with increasing levels of dietary substitution of FO with CSO (*P* < 0.05). Dietary substitution of FO with CSO resulted in changes in the composition of volatile substances in muscle, with 16 volatile substances in muscle significantly affected (*P* < 0.05). The relative expression of genes related to lipid synthesis such as fatty acid synthase (*fas*), acetyl-CoA carboxylase (*acc*) and glycerol-3-phosphate acyltransferase 1 (*gpat1*) in the hepatopancreas were significantly up-regulated in the CSO-50 group compared to other treatment groups (*P* < 0.05). The relative expression of fatty acid anabolism-related genes fatty acyl desaturase 2 (*fads2*) and elongase 4 (*elovl4*) were significantly down-regulated with the increase of dietary substitution of FO with CSO (*P* < 0.05). In conclusion, 50% substitution with CSO had no negative effects on growth performance, promoted lipid synthesis and metabolism, facilitated lipid accumulation. However, complete substitution of FO with CSO inhibited fatty acid synthesis and metabolism, resulting in a lower tissue LC-PUFA content and an altered composition of muscle volatiles.

## 1 Introduction

Fish oil (FO) is renowned for its high content of essential long-chain polyunsaturated fatty acids (LC-PUFA), particularly eicosapentaenoic acid (EPA) and docosahexaenoic acid (DHA) (Maldonado-Othón et al., 2020). It plays a crucial role in the physiological and biochemical processes of aquatic organisms and is a high-quality lipid source in the field of aqua-feed (Li et al., 2023; Yun et al., 2013). With the rapid expansion of aquaculture, global demand for FO has been increasing annually (FAO, 2022). However, due to economic, environmental, and sustainability concerns, more people are looking for suitable alternative oil sources such as olive oil (Toledo et al., 2024), beef tallow (Zhang et al., 2023), camelina and black soldier fly larvae oils (Maldonado-Othón et al., 2022) in order to promote the sustainable development of aquaculture (Hai et al., 2018; Tocher et al., 2019).

Cotton is a primary crop in China, with cottonseed and its processing byproducts serving as significant feed resources (FAO, 2020; Orhevba and Efomah, 2012; Shah, 2017). Cottonseed is one of China's three major oilseed crops. Cottonseed oil can be extracted from cottonseed according to different pre-pressure leaching and refining processes, and the annual output of cottonseed oil (CSO) has exceeded 4 million metric tons in recent years (Liu, 2012; Han et al., 2014). CSO is rich in unsaturated fatty acids and has high nutritional values (Wang et al., 2017; Kumar et al., 2022). At present, CSO, as an energy feed resource, is mainly used in livestock feed, but has been use in aquatic feed, and has not been reported for crustacean use. Free gossypol may be a significant factor limiting the use of CSO in feed (Gadelha et al., 2014; Obert et al., 2007). Nowadays, with the progress and development of production technology, low gossypol or even gossypol free CSO has dominated the CSO market. Evidence suggests that CSO can entirely or partially replace FO as a lipid source for fish. Reports indicate that CSO can serve as the primary dietary lipid source for juvenile gilthead seabream (*Sparus aurata*), with 40 to 48 g/kg of CSO in the diet showing no adverse effects on growth performance and nutritional composition (Wassef et al., 2015). Rainbow trout (*Oncorhynchus mykiss*) showed the highest growth performance and fatty acid composition when the CSO-to-FO ratio was 50% (Güler and Yildiz, 2011). Thus, the prospects for use of CSO in the feed industry are very broad. CSO, as a new alternative lipid source of FO, is urgently needed to demonstrate its potential replacement ability due to a lack of current research.

The swimming crab (*Portunus trituberculatus*) stands as a widely favored marine crustacean in China. It is popular because of its tasty and nutrient rich meat, especially in its content of LC-PUFA (Tocher, 2015; Karalazos, 2022). The composition of volatile substances and fatty acids is an important factor to determine the meat quality and nutritional value of swimming crabs. Lipids and fatty acids serve as precursors of volatile substances. The fatty acid composition of the diet directly affects the fatty acid profiles of the organism. The precursors of volatile substances are lipids and fatty acids. Exogenous intake of lipids in the diet is very important for swimming crabs. Therefore, the objective of present study aims to evaluate the feasibility of replacing FO with CSO in the diet of the swimming crab, in order to provide a theoretical basis for the development of swimming crab feed.

## 2 Materials and methods

### 2.1 Animal ethics statement

All experimental operations involving animals complied with the requirements of the governing regulation for the use of experimental animals in Zhejiang Province (Zhejiang provincial government order No. 263, released on August 17, 2009, effective from October 1, 2010) and Ningbo University Animal Research and Ethics Committee (No. SYXK20190005). The study did not include endangered or protected species.

### 2.2 Experimental diets

Three isonitrogenous (49% crude protein) and isolipidic (12% crude lipid) diets were formulated. Peru fish meal, soybean protein concentrate, soybean meal, krill meal, corn gluten meal and poultry by-product meal were used as protein sources. Wheat flour was used as the dietary carbohydrate source. Cholesterol, FO and CSO were used as the dietary lipid sources. CSO was obtained from Xinjiang Jinlan Plant Protein Co., Ltd. (Xinjiang, China). Diets were formulated replacing FO with CSO at 0%, 50% and 100% (CSO-0, CSO-50, and CSO-100), respectively (Table 1). The free gossypol content in CSO was 27.4 mg/kg, meeting the national standard (≤200 mg/kg, GB/T 5009.148) as confirmed by Zhongke Testing Technology Service Co., Ltd. (Guangzhou, China). The ingredients, proximate composition and fatty acids profiles of experimental diets are shown in Table 1, Table 2. The diets were produced according to the process detailed earlier (Yang et al., 2023). All ingredients were pulverized and ground through an 80-mesh sieve. Ingredients were weighed according to the formulation. The micronutrients (sodium alginate, vitamin and mineral premix) were added to the mix by the step-by-step expansion method. FO, CSO and soybean lecithin were poured in and mixed thoroughly, followed by mixing with a Hobart-type mixer. During this period, choline chloride and Ca(H_2_PO_4_)_2_ were dissolved in ultrapure water weighing approximately 50% of the total weight of the ingredients and added to the mixer for blending. Finally, the mixture was extruded into strips using a twin-screw extruder (F-26, South China University of Technology, Guangzhou, China) and pelletized into feeds with particle sizes of 3 and 5 mm and lengths of 0.8 to 1.3 cm using a pelletizer (G-250, South China University of Technology, Guangzhou, China). The feeds were air-dried and kept at −20 °C until use.

**Table 1:** Composition and nutrient levels of the experimental diets (DM basis, %).

Item	Substitution of FO with CSO, %		
	0	50	100
Ingredients			
Peru fish meal^1^	20.00	20.00	20.00
Soybean protein concentrate^1^	20.00	20.00	20.00
Soybean meal^1^	15.00	15.00	15.00
Krill meal^1^	2.00	2.00	2.00
Corn gluten meal^1^	3.00	3.00	3.00
Poultry by-product meal^1^	8.00	8.00	8.00
Wheat flour^1^	19.50	19.50	19.50
Soybean lecithin^1^	1.00	1.00	1.00
Cholesterol^2^	0.50	0.50	0.50
Fish oil^1^	5.00	2.50	
CSO^3^	0.00	2.50	5.00
Vitamin premix^4^	0.50	0.50	0.50
Mineral premix^4^	1.00	1.00	1.00
Ca(H_2_PO_4_)_2_	2.00	2.00	2.00
Choline chloride	0.30	0.30	0.30
Sodium alginate	2.00	2.00	2.00
Butylated hydroxytoluene	0.20	0.20	0.20
Total	100.00	100.00	100.00
Nutrient levels^5^			
DM	93.93	92.63	94.78
CP	45.32	45.31	45.23
CL	12.02	11.68	11.16
Ash	9.41	9.33	9.21

FO = fish oil; CSO = cottonseed oil; CP = crude protein; CL = crude lipids; DM = dry matter.

1 Ningbo Tech-Bank Feed Co., Ltd. Ningbo, China. Peru fish meal (CP: 70.77% DM, CL: 8.78% DM); soybean protein concentrate (CP: 69.09% DM, CL: 0.43% DM); soybean meal (CP: 51.72% DM, CL: 1.06% DM); krill meal (CP: 54.33% DM, CL: 21.13% DM); corn gluten meal (CP: 60.12% DM, CL: 0.97% DM); poultry by-product meal (CP: 73.76% DM, CL:10.46% DM); wheat flour (CP:16.12% DM, CL:1.41% DM).

2 Shanghai Macklin Biochemical Co., Ltd., China. Cholesterol, 99%.

3 Xinjiang Jinlan Plant Protein Co., Ltd., China.

4 Vitamin premix and mineral premix were based on Jin et al. (2013). Vitamin premix (per kilogram premix): retinyl acetate 2,500,000 IU, cholecalciferol 500,000 IU, all-rac-a-tocopherol 25,000 IU, menadione 5.63 g, thiamine 11.25 g, riboflavin 9.5 g, ascorbic acid 95 g, pyridoxine hydrochloride 10 g, cyanocobalamin 0.02 g, folic acid 2 g, biotin 0.375 g, nicotinic acid 37.5 g, D-Ca pantothenate 21.5 g, inositol 80 g, antioxidant 0.5 g, antioxidant corn starch 696.775 g. Mineral premix (g/kg premix): FeC_6_H_5_O_7_ 4.57, ZnSO_4_·7H_2_O 9.43, MnSO_4_·H_2_O (99%) 4.14, CuSO_4_·5H_2_O (99%) 6.61, MgSO_4_·7H_2_O (99%) 238.97, CoCl_2_·6H_2_O (99%) 1.36.

5 The nutrient levels were measured.

**Table 2:** The fatty acid composition of the experimental diets (DM basis, mg/g).

Item	Substitution of FO with CSO, %		
	0	50	100
14:0	4.90	3.64	2.29
16:0	18.78	19.36	19.37
18:0	4.16	3.74	3.20
20:0	0.34	0.30	0.25
∑SFA	28.18	27.05	25.12
16:1n	5.20	3.65	2.11
18:1n-9	18.90	19.61	20.35
20:1n-9	1.27	0.72	0.21
22:1n-11	0.19	0.13	0.08
∑MUFA	25.56	24.12	22.76
18:2n-6	5.80	11.68	16.74
18:3n-6	0.10	0.07	0.08
20:2n-6	0.13	0.10	0.06
20:4n-6	0.90	0.66	0.42
22:4n-6	0.15	0.12	0.09
∑n-6 PUFA	7.08	12.64	17.39
18:3n-3	1.72	1.53	1.31
18:4n-3	0.96	0.66	0.39
20:4n-3	0.33	0.20	0.09
20:5n-3	7.13	5.21	3.43
22:5n-3	0.99	0.74	0.49
22:6n-3	9.13	5.87	3.08
∑n-3 PUFA	20.26	14.22	8.80
∑LC-PUFA	17.16	11.74	6.93
n-3/n-6 PUFA	2.86	1.12	0.51
DHA/EPA	1.28	1.13	0.90

DM = dry matter; FO = fish oil; CSO = cottonseed oil; ∑SFA = total saturated fatty acids; ∑MUFA = total mono-unsaturated fatty acids; ∑n-6PUFA = total n-6 polyunsaturated fatty acids; ∑n-3PUFA = total n-3 polyunsaturated fatty acids; ∑LC-PUFA = total long-chain polyunsaturated fatty acids; PUFA = polyunsaturated fatty acids; DHA/EPA = 22:6n-3/20:5n-3.

### 2.3 Feeding trial

Juvenile swimming crabs were acquired from a commercial farm in Xiangshan (Ningbo, China), and feeding trials were carried out by Ningbo Xiangshan Harbor Seed Co., Ltd. (Ningbo, China). Domestication and acclimation with commercial feed (45% crude protein and 8% crude lipid, Ningbo Tech-Bank Corp., Ningbo, China) were performed for two weeks before to the feeding experiment. Ninety healthy juvenile swimming crabs, initially weighing 34.28 ± 0.59 g each, were allocated to 90 marked plastic baskets and distributed across three pools. Each dietary regimen was randomly allocated to three replicates, with each replicate comprising a cohort of 10 crabs. During the six-week feeding phase, swimming crabs were fed daily at 17:30 until they appeared satiated. The seawater temperature in pools was 26.00 to 28.00 °C, with a salinity of 23.50‰ to 24.50‰, dissolved oxygen content of 6.90 to 8.10 mg/L, and ammonia nitrogen content of 0.03 mg/L.

### 2.4 Sample collection

At the end of the feeding trial, the number of surviving crabs and the final body weight were recorded for each plastic basket. Five crabs per replicate were sampled for hemolymph, and the supernatant was separated by centrifugation (4 °C, 1269.8 × *g*, 10 min) and stored at −80 °C. Crabs were dissected for each replicate, and one crab's fresh hepatopancreatic tissue was taken and preserved in 4% paraformaldehyde for paraffin section preparation. Hepatopancreas and muscles were collected and instantly frozen in liquid nitrogen before being preserved at −80 °C for further examination.

### 2.5 Chemical analysis

#### 2.5.1 Proximate composition and fatty acid profiles in diets, hepatopancreas and muscles

The proximate composition of dietary ingredients, experimental diets, and tissues was ascertained through the application of standard methods as stipulated by the Association of Official Analytical Chemists (AOAC) in the year 2006 (AOAC, 2006). To assess the moisture content, samples were dried to a constant weight at 105 °C (method 934.01). Crude protein content was measured using the dumas combustion method and a protein analyzer (FP-528, Leco, USA) (method 968.06). The crude lipid contents were determined using the ether extraction method with a Soxtec System HT (Tecator, Sweden) (method 920.39). The ash content was measured by heating the samples to 550 °C in a muffle furnace until they reached a consistent weight (method 942.05). The methodology used for analyzing fatty acid profiles in both diets and tissues were consistent with those outlined in a recent scientific paper (Yang et al., 2023).

#### 2.5.2 Hemolymph biochemical indices and hepatopancreatic lipid-related enzyme activities

All hemolymph biochemical assays, including albumin (ALB), alkaline phosphatase (ALP), glucose (GLU), aspartate aminotransferase (AST), alanine amino transferase (ALT) and lipid composition such as total cholesterol (T-CHO), high-density lipoprotein cholesterol (HDL-C), low-density lipoprotein cholesterol (LDL-C), and triglyceride (TAG) were performed using an automated biochemical analyzer (VITALAB SELECTRA Junior Pros, Netherlands). Non esterified free fatty acids (NEFA) in the hemolymph and hepatopancreas, as well as the lipid composition (T-CHO, HDL-C, LDL-C, TAG) in the hepatopancreas, were quantified following the guidelines provided by commercial assay kits (Jian Cheng Bioengineering Institute, Nanjing, China).

#### 2.5.3 Histological analysis of hepatopancreas

Hepatopancreas samples fixed with 4% paraformaldehyde were subjected to paraffin preparation at Hulk Biotechnology Company (Hangzhou, China). The sample was pruned and then progressively dehydrated in 75% to 100% ethanol. After that, the sample was embedded in paraffin wax and cut into slices about 4 μm in size. Hematoxylin and eosin (H&E) were used for staining. Finally, hepatopancreas were observed and photographed under a light microscope (Nikon Eclipse CI, Tokyo, Japan).

#### 2.5.4 Detection of volatile substances composition

The determination of volatile substances in muscle samples was performed by headspace solid phase microextraction (HS-SPME) based on the method proposed by Yuan et al. (2020), with certain modifications. The pre-treatment steps were mainly as follows: 1) muscle samples were thawed on ice, after which sterile scissors were used to cut the muscle samples into approximately 3-g samples which were placed in a 20-mL headspace bottle (CNW, Germany), ensuring they did not stick to the bottle wall and were positioned at the bottom; 2) 2 mL of saturated sodium chloride solution and 3 μL internal standard of 2,4,6-trimethylpyridine (TMP, 99%, Sigma–Aldrich, Shanghai, China) was added to the headspace bottle, and stirred gently; 3) samples were refrigerated at 4 °C before machine testing.

Analysis was performed using 7890B–7000C gas chromatography-mass spectrometry (GC-MS, Agilent Technologies, USA). The system included an automatic solid phase microextraction sampling system (Gerstel, Germany, 50/30 μm DVB/CAR/PDMS), a solid phase microextraction head (Supelco, USA), and a VOCOL capillary column (60.00 m × 0.32 mm × 1.80 μm, CNW, Germany). Volatile compound content was expressed as relative content (ng/g) using the formula:

Volatile compound content = [(measured compound peak area/TMP peak area) × 1 μg TMP]/muscle sample weight.

### 2.6 Total RNA extraction and real time quantitative PCR (RT-qPCR)

Total RNA extraction from the hepatopancreas samples was carried out using Trizol reagent (Vazyme, Biotech Co., Ltd., Nanjing, China). The assessment of isolated total RNA quality was conducted using a NanoDrop 2000 (Thermo Fisher Scientific, USA). Only high-quality RNA samples, characterized by an A260/A280 ratio falling within the range of 1.8 to 2.0, were considered for subsequent processing. The reverse transcription of total RNA into cDNA template was achieved using the HiScript II Reverse Transcriptase Kit (Vazyme Biotech Co., Ltd., Nanjing, China). Gene-specific primers for RT-qPCR were designed using Primer Premier 5.0 (Table 3). All primers utilized in this investigation were synthesized by Beijing Tsingke Biotech Co., Ltd. (Beijing, China), with β-actin serving as the designated reference gene. RT-qPCR was conducted utilizing ChamQ SYBR qPCR Green Master Mix (Vazyme) in the quantitative thermal cycler (Lightcycler 96, Roche, Switzerland), adhering to established procedural protocols (Yang et al., 2022). Briefly, samples were heated to 95 °C for 2 min, followed by 45 cycles of 95 °C for 10 s, 58 °C for 10 s and 72 °C for 20 s. The relative expression of genes was determined using the delta-data CT method (2^−ΔΔCt^) (Livak and Schmittgen, 2001). Each treatment condition was conducted in triplicate.

**Table 3:** The sequences of primers in this study.

Gene	Forward (5′ to 3′)	Reverse (5′ to 3′)	Size, bp	Accession number
β-actin	CGAAACCTTCAACACTCCCG	GGATAGCGTGAGGAAGGGCATA	609	FJ641977.1
*fas*	CTTCAATACCCACCAAACC	CCTCAATGATGCCAGACAC	299	MF537400
*acc*	TCTCAGGGCAACCTTACGCT	CGGGAGGCAGTAACCATTCA	293	MF537401
*g6pd*	TGAAAAGGTGAAGGTGCTGA	CGGTGGAGTCATCAAGGTAAC	125	MF537402
*6pgd*	GGGTGGAACCTCAACTATGG	CGATAGCCATCATAGAAAGCC	254	MF537403
*dgat1*	TGGCGTCTCTGGAACCTACC	CATCAAGTTACCAATGCGGG	258	MF537404
*gpat1*	TCATTGAAGGAGGACGAAC	GCTTTGTCCCATCTGTTCC	179	PRJNA432636
*gpat3*	GGGACCGAGCACAAGTTATT	GCGATGGGGTAGATGACTGT	160	PRJNA432636
*hnf4α*	CCTGTATCAAAGCCATCGT	CGCTGGAAGGGTTAGAAGA	170	PRJNA432636
*rxr*	CTCAAGGCTGGCTGGAATG	CCTTTGGCATCTGGGTTGA	245	PRJNA432636
*fads2*	GCAGTGAGAGACAGGACGGA	CTGGATGGTTAGGGTTTGGG	241	PRJNA432636
*elovl4*	AGCTACACAGGATGAAGGACC	GAGCAGCATAATGGCAAGG	215	PRJNA432636
*srebp1*	GTGATGTGTGCCTTGCGAGT	CCAGGGTTCACCAGTGTAGT	284	MF537405
*fabp1*	CACTCGCCAGTAGTCAATAGG	TCACTTAGAGAGCAAAGGTCAC	219	KU950355
*fabp3*	GAAGGCACTTGGTGTTGGA	TCTTGAGGGTGGAGATGGT	119	PRJNA432636
*fabp4*	AAGAATGACCCAATGCGTG	GCCAGCGAAAGGTGTCTC	178	PRJNA432636
*cpt1*	GCTTGCCTACTACCGACAC	CCTTGGACATCTTACTGCTC	155	MF537407
*cpt2*	TGGGACAAGGTTTTGATAGGC	TGGAGATGATGATGTGGTTGA	123	PRJNA432636
*acox2*	ACCAACCACGGCATTCAC	TTGTCCACCCCATTCAGC	116	PRJNA432636

*fas* = fatty acid synthase; *acc* = acetyl-CoA carboxylase; *g6pd* = glucose 6-phosphate dehydrogenase; *6pgd* = 6-phosphogluconate dehydrogenase; *dgat1 =* diacylglycerol acyltransferase 1; *gpat1 =* glycerol-3-phosphate acyltransferase 1; *gpat3 =* glycerol-3-phosphate acyltransferase 3; *hnf4α* = hepatocyte nuclear factor 4-alpha; *rxr* = retinoid X receptor; *fads2* = fatty acyl desaturase 2; *elovl4* = elongase4; *srebp1* = sterol regulatory element-binding protein 1; *fabp1* = fatty acid binding protein 1; *fatp3* = fatty acid transport proteins 3; *fatp4* = fatty acid transport proteins 4; *cpt1* = carnitine palmitoyltransferase 1; *cpt3* = carnitine palmitoyltransferase *3*; *acox2* = acyl-CoA oxidase 2.

### 2.7 Calculations and statistical analysis

The parameters in this study were calculated as follows:

Percent weight gain (PWG, %) = 100 × (*W*_1_ – *W*_2_)/*W*_2_;

Specific growth rate (SGR, %/d) = 100 × (Ln*W*_1_ – Ln*W*_2_)/days;

Survival (%) = 100 × final fish number/initial fish number;

Feed conversion ratio (FCR) = dry feed consumed/(*W*_1_ − *W*_2_);

Feed intake (FI, %/d) = 100 × total feed intake (g)/[(*W*_1_ + *W*_3_ - *W*_2_) × days],

Where *W*_1_ and *W*_2_ are the final body weight and initial body weight, *W*_3_ is the dead crab weight, respectively.

Statistical analyses were conducted using SPSS 19.0 software, with results expressed as means ± standard error of the mean (SEM). Data sets were analyzed via one-way analysis of variance (ANOVA), followed by Tukey's multiple range test for post hoc analysis, with statistical significance set at a *P-*value of less than 0.05. Linear and quadratic relationships were established. Principal component analysis (PCA), hierarchical cluster analysis (HCA) and heatmap visualization (HMV) of hepatopancreas and muscle fatty acid composition of swimming crabs were performed according to previously reported methods (Yang et al., 2023).

## 3 Results

### 3.1 Growth performance and survival

The effects of dietary CSO substitution for FO on growth performance and survival of the swimming crabs are presented in Table 4. Crabs fed with the CSO-100 diet exhibited lower PWG, SGR and survival than those fed with the CSO-0 and CSO-50 diets (*P* < 0.05). FCR and FI were not significantly affected by the dietary CSO substitution for FO (*P* > 0.05).

**Table 4:** Effects of dietary substitution of FO with CSO on the growth performance and survival for swimming crabs.

Items	Substitution of FO with CSO, %			*P-*value		
	0	50	100	ANOVA	Linear	Quadratic
Initial body weight, g	34.87 ± 0.789	34.04 ± 0.520	33.91 ± 0.159	0.462	0.266	0.621
Percent weight gain, %	134.14 ± 3.291^b^	124.69 ± 4.653^b^	106.17 ± 2.763^a^	0.003	0.001	0.306
Specific growth rate, %/d	1.48 ± 0.032^b^	1.44 ± 0.038^b^	1.29 ± 0.023^a^	0.003	0.002	0.018
Survival, %	72.73 ± 3.090^b^	72.73 ± 3.248^b^	60.61 ± 2.123^a^	0.006	0.003	0.135
Feed conversion ratio	2.38 ± 0.403	2.40 ± 0.330	2.46 ± 0.329	0.986	0.792	0.939
Feed intake, %/d	3.22 ± 0.148	3.38 ± 0.078	3.55 ± 0.278	0.488	0.250	0.983

FO = fish oil; CSO = cottonseed oil.

^a,b^Means in the same row with different letter superscripts show significant differences (*P* < 0.05). Data was provided as mean ± SEM of three replications (*n* = 3).

### 3.2 Biochemical indices in the hemolymph

The effects of dietary CSO substitution for FO on hemolymph biochemical indices are shown in Table 5. Crabs fed with the CSO-100 diet showed lower concentration of ALB and ALP in hemolymph than those fed with the CSO-0 and CSO-50 diets (*P* < 0.05). However, crabs fed diet with CSO-50 exhibited the lowest ALT activity in hemolymph among all the treatment groups (*P ＜* 0.001). GLU, T-CHO, LDL-C, TAG, and NEFA in hemolymph were significantly influenced by dietary CSO substitution for FO (*P* < 0.05). Crabs fed diet with CSO-0 had lower concentration of NEFA in hemolymph than those other treatment groups (*P* = 0.014).

**Table 5:** Effects of dietary substitution of FO with CSO on hemolymph biochemical indices for swimming crabs.

Items	Substitution of FO with CSO, %			*P-*value		
	0	50	100	ANOVA	Linear	Quadratic
ALB, g/L	4.92 ± 0.019^c^	4.48 ± 0.001^b^	4.04 ± 0.043^a^	＜0.001	＜0.001	0.954
ALP, U/L	12.87 ± 1.485^b^	10.56 ± 0.131^b^	9.75 ± 0.304^a^	0.005	0.005	0.273
GLU, g/L	1.65 ± 0.093^a^	1.59 ± 0.017^a^	2.03 ± 0.100^b^	0.015	0.015	0.042
AST, U/L	227.07 ± 14.596	211.29 ± 6.119	224.57 ± 0.599	0.469	0.853	0.242
ALT, U/L	109.38 ± 0.576^c^	87.42 ± 0.595^a^	99.86 ± 1.169^b^	＜0.001	＜0.001	＜0.001
T-CHO, mmol/L	0.54 ± 0.014^a^	0.65 ± 0.012^b^	0.49 ± 0.020^a^	0.001	0.039	＜0.001
HDL-C, mmol/L	0.21 ± 0.000	0.20 ± 0.001	0.21 ± 0.001	0.890	0.699	0.256
LDL-C, mmol/L	0.24 ± 0.007^a^	0.27 ± 0.002^b^	0.22 ± 0.001^a^	0.001	0.026	＜0.001
TAG, mmol/L	0.24 ± 0.001^b^	0.25 ± 0.001^b^	0.22 ± 0.001^a^	＜0.001	＜0.001	＜0.001
NEFA, mmol/L	44.71 ± 1.359^a^	66.80 ± 8.257^b^	70.96 ± 7.943^b^	0.014	0.003	0.114

FO = fish oil; CSO = cottonseed oil; ALB = albumin; ALP = alkaline phosphatase; GLU = glucose; AST = aspartate aminotransferase; ALT = alanine amino transferase; T-CHO = total cholesterol; HDL-C = high-density lipoprotein cholesterol; LDL-C = low-density lipoprotein cholesterol; TAG = triglyceride; NEFA = non esterified free fatty acids.

^a-c^ Means in the same row with different letter superscripts show significant differences (*P* < 0.05). Data was provided as mean ± SEM of three replications (*n* = 3).

### 3.3 Effect of CSO as a substitute for FO on the hepatopancreas

#### 3.3.1 Lipid composition

The effects of dietary substitution of FO with CSO on hepatopancreatic lipid composition in the swimming crabs are presented in Table 6. Crabs fed with the CSO-50 diet exhibited higher hepatopancreatic T-CHO content than those fed with the CSO-0 and CSO-100 diets, the lowest hepatopancreatic HDL-C content was observed at crabs fed with the CSO-50 diet (*P* < 0.05). Crabs fed with the CSO-100 diet showed significantly higher TAG and NEFA contents in hepatopancreas than those other treatment groups (*P* < 0.05).

**Table 6:** Effects dietary substitution of FO with CSO on lipid composition (mmol/mg prot) in the hepatopancreas for swimming crabs.

Items	Substitution of FO with CSO, %			*P-*value		
	0	50	100	ANOVA	Linear	Quadratic
T-CHO	0.02 ± 0.000^a^	0.03 ± 0.001^b^	0.02 ± 0.002^a^	0.001	0.911	＜0.001
HDL-C	0.67 ± 0.034^b^	0.50 ± 0.017^a^	0.64 ± 0.007^b^	0.003	0.326	0.001
LDL-C	0.02 ± 0.001	0.02 ± 0.003	0.02 ± 0.001	0.609	0.555	0.438
TAG	0.12 ± 0.010^a^	0.10 ± 0.006^a^	0.17 ± 0.009^b^	0.004	0.008	0.006
NEFA	214.95 ± 21.362^a^	266.79 ± 3.017^a^	377.52 ± 5.272^b^	＜0.001	＜0.001	0.110

FO = fish oil; CSO = cottonseed oil; T-CHO = total cholesterol; HDL-C = high-density lipoprotein cholesterol; LDL-C = low-density lipoprotein cholesterol; TAG = triglyceride; NEFA = non esterified free fatty acids.

^a，b^Means in the same row with different letter superscripts show significant differences (*P* < 0.05). Data was provided as mean ± SEM of three replications (*n* = 3).

#### 3.3.2 Lipid content

The effects of dietary substitution of FO with CSO on the lipid content in hepatopancreas of the swimming crabs are shown in Table 7. The highest lipid content in hepatopancreas was occurred at carbs fed with CSO-50 diet, however, crabs fed diet with CSO-0 showed lower lipid content in hepatopancreas than those fed with CSO-50 and CSO-100 diets (*P* = 0.005).

**Table 7:** Effects of dietary substitution of FO with CSO on lipid content (g/100 g) in hepatopancreas of swimming crabs.

Item	Substitution of FO with CSO, %			*P-*value		
	0	50	100	ANOVA	Linear	Quadratic
Lipid	31.32 ± 0.915^a^	40.01 ± 0.011^c^	36.26 ± 1.714^b^	0.005	0.021	0.004

FO = fish oil; CSO = cottonseed oil.

^a-c^ Means in the same row with different letter superscripts show significant differences (*P* < 0.05). Data was provided as mean ± SEM of three replications (*n* = 3).

#### 3.3.3 Histological structure

**Fig. 1:**
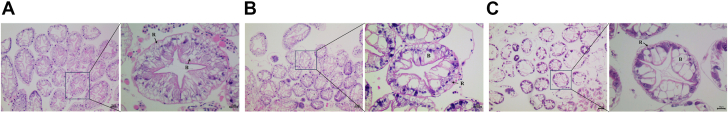
Effects of dietary substitution of fish oil (FO) with cottonseed oil (CSO) on histological structure in the hepatopancreas of the swimming crabs. A-C: hematoxylin and eosin (H&E) staining sections of hepatopancreas at 0% to 100% CSO replacement level, respectively. Enlarged 400 ×, bar = 20 μm, B represents blister-like cell and R represents resorptive cell.

The effects of dietary substitution of FO with CSO on hepatopancreatic histological structure of the swimming crabs are presented in Fig. 1. The number of blister-like (B) cells in the hepatopancreas increased with increased CSO substitution. However, the number of resorptive (R) cells showed a decreasing trend when CSO substitution increased from 0% to 100%.

#### 3.3.4 Fatty acid profiles

The effects of dietary substitution of FO with CSO on fatty acid composition in the hepatopancreas are shown in Table 8. The contents of total saturated fatty acids (SFA), total mono-unsaturated fatty acids (MUFA), and total long-chain poly-unsaturated fatty acids (LC-PUFA) in the hepatopancreas decreased significantly with the increased of dietary substitution of FO with CSO (*P* < 0.05). However, linoleic acid (18:2n-6) and total n-6PUFA contents in hepatopancreas significantly increased when dietary substitution of FO with CSO increased from 0% to 100% (*P* < 0.05).

**Table 8:** Effects of dietary substitution of FO with CSO on fatty acid composition (mg/g) in the hepatopancreas for swimming crabs.

Items	Substitution of FO with CSO, %			*P-*value		
	0	50	100	ANOVA	Linear	Quadratic
14:0	7.68 ± 0.411^c^	5.51 ± 0.078^b^	3.54 ± 0.194^a^	＜0.001	＜0.001	0.770
16:0	47.65 ± 0.606^b^	42.76 ± 0.538^a^	40.83 ± 0.633^a^	＜0.001	＜0.001	0.088
18:0	15.06 ± 0.354^a^	17.52 ± 0.243^b^	17.21 ± 0.161^b^	0.001	0.001	0.005
20:0	1.27 ± 0.039^b^	1.13 ± 0.051^b^	0.94 ± 0.005^a^	0.002	0.001	0.688
∑SFA	75.77 ± 1.896^b^	66.92 ± 0.909^a^	62.51 ± 0.984^a^	0.001	＜0.001	0.224
16:1n	10.36 ± 0.331^c^	4.86 ± 0.125^b^	2.69 ± 0.140^a^	＜0.001	＜0.001	0.001
18:1n-9	62.11 ± 0.331^b^	48.87 ± 0.125^a^	46.39 ± 0.140^a^	＜0.001	＜0.001	＜0.001
20:1n-9	4.56 ± 0.111^c^	2.38 ± 0.016^b^	1.13 ± 0.015^a^	＜0.001	＜0.001	0.001
22:1n-11	0.72 ± 0.017^c^	0.42 ± 0.006^b^	0.34 ± 0.018^a^	＜0.001	＜0.001	0.001
∑MUFA	77.75 ± 0.960^c^	56.53 ± 0.906^b^	50.53 ± 0.548^a^	＜0.001	＜0.001	＜0.001
18:2n-6	12.99 ± 0.064^a^	20.73 ± 0.239^b^	28.35 ± 1.048^c^	＜0.001	＜0.001	0.937
20:2n-6	2.81 ± 0.091^a^	4.12 ± 0.070^b^	5.27 ± 0.102^c^	＜0.001	＜0.001	0.490
20:4n-6	2.76 ± 0.039^c^	1.90 ± 0.053^b^	1.50 ± 0.004^a^	＜0.001	＜0.001	0.003
22:4n-6	0.32 ± 0.020^b^	0.26 ± 0.026^ab^	0.22 ± 0.003^a^	0.035	0.013	0.813
∑n-6 PUFA	19.46 ± 0.124^a^	27.52 ± 0.338^b^	35.75 ± 0.970^c^	＜0.001	＜0.001	0.911
18:3n-3	2.49 ± 0.148^b^	1.70 ± 0.040^a^	1.56 ± 0.065^a^	0.001	＜0.001	0.035
18:4n-3	0.76 ± 0.024^c^	0.47 ± 0.018^b^	0.25 ± 0.005^a^	＜0.001	＜0.001	0.197
20:4n-3	0.80 ± 0.024^c^	0.37 ± 0.011^b^	0.26 ± 0.023^a^	＜0.001	＜0.001	0.001
20:5n-3	11.71 ± 0.094^c^	9.54 ± 0.266^b^	8.53 ± 0.020^a^	＜0.001	＜0.001	0.027
22:5n-3	2.68 ± 0.072^c^	1.59 ± 0.051^b^	1.16 ± 0.011^a^	＜0.001	＜0.001	0.002
22:6n-3	23.25 ± 0.52^c^	14.07 ± 0.29^b^	9.00 ± 0.06^a^	＜0.001	＜0.001	0.003
∑n-3 PUFA	42.80 ± 1.242^c^	27.75 ± 0.672^b^	20.76 ± 0.065^a^	＜0.001	＜0.001	0.007
∑LC-PUFA	37.54 ± 0.183^c^	25.51 ± 0.606^b^	19.03 ± 0.043^a^	＜0.001	＜0.001	0.001
n3/n6 PUFA	2.29 ± 0.079^c^	1.00 ± 0.013^b^	0.54 ± 0.022^a^	＜0.001	＜0.001	＜0.001
DHA/EPA	1.85 ± 0.079^c^	1.50 ± 0.013^b^	1.05 ± 0.022^a^	＜0.001	＜0.001	0.019

FO = fish oil; CSO = cottonseed oil; ∑SFA = total saturated fatty acids; ∑MUFA = total mono-unsaturated fatty acids; ∑n-6PUFA = total n-6 polyunsaturated fatty acids; ∑n-3PUFA = total n-3 polyunsaturated fatty acids; ∑LC-PUFA = total long-chain polyunsaturated fatty acids; PUFA = polyunsaturated fatty acids; DHA/EPA = 22:6n-3/20:5n-3.

^a-c^ Means in the same row with different letter superscripts show significant differences (*P* < 0.05). Data was provided as mean ± SEM of three replications (*n* = 3).

**Fig. 2:**
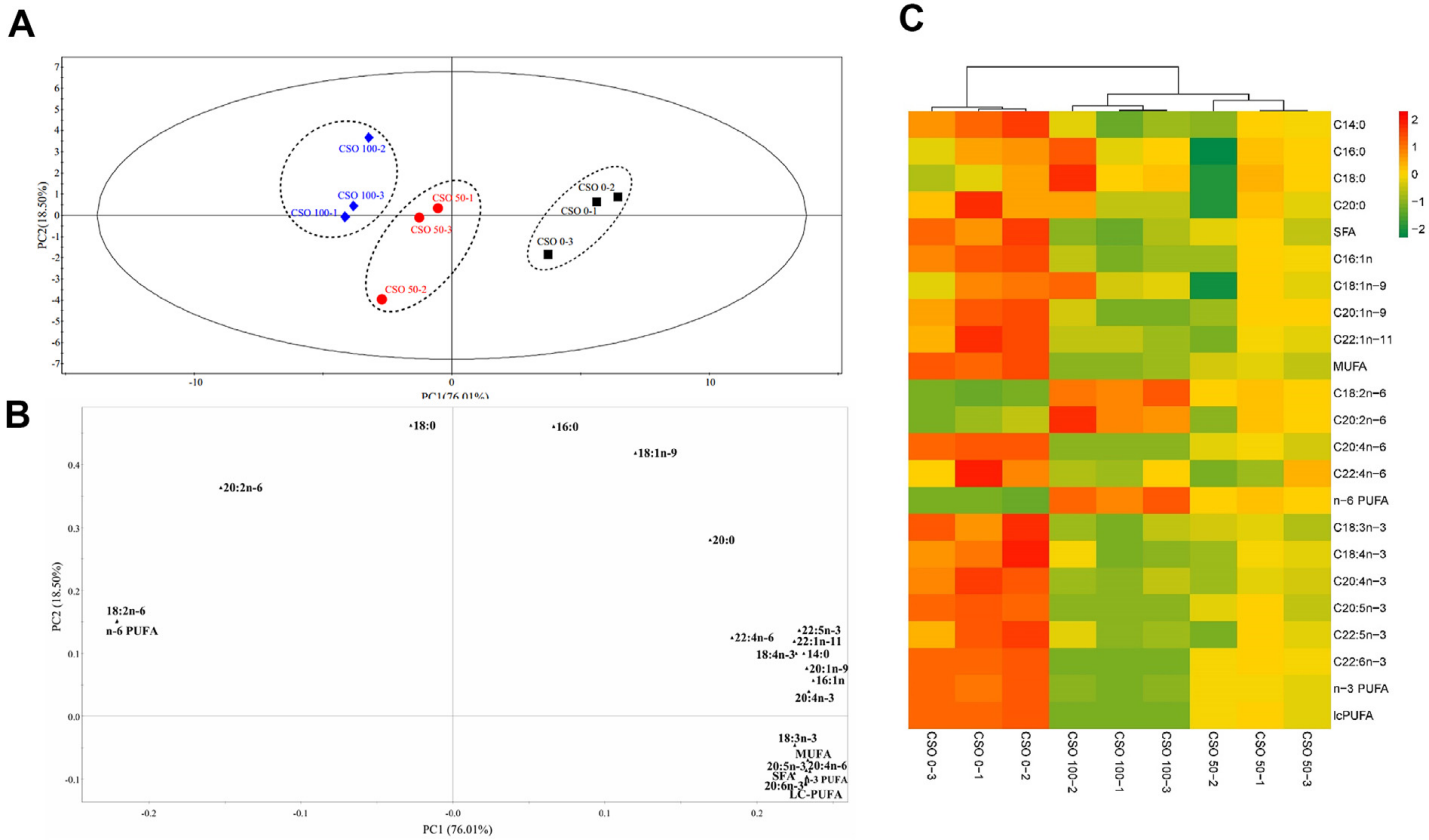
Principal component analysis (PCA) modeling of hepatopancreas fatty acids data (A and B); visualized heat map of hepatopancreas fatty acids data (C). CSO 0-1: substitution of fish oil with 0% cottonseed oil - 1; CSO 0-2: substitution of fish oil with 0% cottonseed oil - 2; CSO 0-3: substitution of fish oil with 0% cottonseed oil - 3; CSO 50-1: substitution of fish oil with 50% cottonseed oil - 1; CSO 50-2: substitution of fish oil with 50% cottonseed oil - 2; CSO 50-3: substitution of fish oil with 50% cottonseed oil - 3; CSO 100-1: substitution of fish oil with 100% cottonseed oil - 1; CSO 100-2: substitution of fish oil with 100% cottonseed oil - 2; CSO 100-3: substitution of fish oil with 100% cottonseed oil – 3. SFA = saturated fatty acids; MUFA = mono-unsaturated fatty acids; n-6PUFA = n-6 polyunsaturated fatty acids; n-3PUFA = n-3 polyunsaturated fatty acids; LC-PUFA = long-chain polyunsaturated fatty acids; DHA/EPA = 22:6n-3/20:5n-3.

As shown in Fig. 2A and B, according to the PCA score plot alongside the fatty acid compositions in the hepatopancreas, the first two main components (PC) accounted for 94.51% of the variance, with individual contributions of 76.01% and 18.50% (Fig. 2A). The score plot showed three separate clusters, organized from right to left, representing dietary categories with CSO replacement levels ranging from 0% to 100%. Figure 2B demonstrates that linolenic acid (18:3n-3, ALA), arachidonic acid (20:4n-6, ARA), and ∑LC-PUFA were found on the bottom right side of PC1 which were quite similar to the control group.

As shown in Fig. 2C, according to the heat maps for Pearson's correlation analysis in the hepatopancreas, the highest fatty acid contents and a single cluster appeared in the CSO-0 group. CSO-50 and CSO-100 groups had similar fatty acid composition.

### 3.4 Effect of CSO as a substitute for FO on the muscle

#### 3.4.1 Lipid content

As shown in Table 9, the muscle lipid content significantly decreased with increasing dietary substitution of FO with CSO (*P* = 0.021).

**Table 9:** Effects of dietary substitution of FO with CSO on lipid content (g/100 g) in muscle of swimming crabs.

Item	Substitution of FO with CSO, %			*P-*value		
	0	50	100	ANOVA	Linear	Quadratic
Lipid	1.66 ± 0.091^b^	1.22 ± 0.167^ab^	1.07 ± 0.024^a^	0.021	0.009	0.324

FO = fish oil; CSO = cottonseed oil.

^a,b^ Means in the same row with different letter superscripts show significant differences (*P* < 0.05). Data was provided as mean ± SEM of three replications (*n* = 3).

#### 3.4.2 Fatty acid profiles

The effects of dietary substitution of FO with CSO on fatty acid composition in the muscle are shown in Table 10. LA content in muscle were significantly increased with the increasing CSO supplementation (*P* < 0.001). The contents of ALA, eicosapentaenoic acid (20:5n-3, EPA), docosahexaenoic acid (22:6n-3, DHA), and ARA exhibited a noteworthy decline when dietary substitution of FO with CSO increased from 0% to 100% (*P* < 0.05).

**Table 10:** Effects of dietary substitution of FO with CSO on fatty acid composition (mg/g) in the muscle for swimming crabs.

Items	Substitution of FO with CSO, %			*P-*value		
	0	50	100	ANOVA	Linear	Quadratic
14:0	0.34 ± 0.015^b^	0.17 ± 0.001^a^	0.20 ± 0.007^a^	＜0.001	＜0.001	＜0.001
16:0	5.72 ± 0.008^c^	4.92 ± 0.046^a^	5.40 ± 0.030^b^	＜0.001	＜0.001	＜0.001
18:0	3.16 ± 0.038^b^	2.99 ± 0.008^a^	3.33 ± 0.050^c^	0.002	0.020	0.001
20:0	0.08 ± 0.011	0.06 ± 0.003	0.06 ± 0.004	0.188	0.124	0.299
∑SFA	9.29 ± 0.058^c^	8.14 ± 0.035^a^	8.94 ± 0.028^b^	＜0.001	0.001	＜0.001
16:1n	0.50 ± 0.008^b^	0.25 ± 0.006^a^	0.23 ± 0.012^a^	＜0.001	＜0.001	＜0.001
18:1n-9	6.75 ± 0.044^c^	5.26 ± 0.014^a^	5.50 ± 0.007^b^	＜0.001	＜0.001	＜0.001
20:1n-9	0.12 ± 0.005^b^	0.07 ± 0.008^a^	0.05 ± 0.003^a^	0.001	＜0.001	0.135
22:1n-11	0.06 ± 0.010	0.06 ± 0.004	0.08 ± 0.008	0.327	0.201	0.451
∑MUFA	7.07 ± 0.034^c^	5.64 ± 0.012^a^	5.86 ± 0.002^b^	＜0.001	＜0.001	＜0.001
18:2n-6	1.97 ± 0.145^a^	2.63 ± 0.045^b^	3.63 ± 0.068^c^	＜0.001	＜0.001	0.201
20:2n-6	0.26 ± 0.001^a^	0.31 ± 0.002^b^	0.43 ± 0.010^c^	＜0.001	＜0.001	0.001
20:4n-6	0.70 ± 0.032	0.71 ± 0.012	0.69 ± 0.015	0.817	0.668	0.659
∑n-6 PUFA	3.02 ± 0.111^a^	3.66 ± 0.025^b^	4.76 ± 0.035^c^	＜0.001	＜0.001	0.035
18:3n-3	0.22 ± 0.008^b^	0.17 ± 0.009^a^	0.14 ± 0.005^a^	0.001	＜0.001	0.001
18:4n-3	0.05 ± 0.002^b^	0.02 ± 0.002^a^	0.02 ± 0.001^a^	＜0.001	＜0.001	0.001
20:4n-3	0.07 ± 0.005^b^	0.06 ± 0.003^a^	0.05 ± 0.001^a^	0.004	0.001	0.624
20:5n-3	5.84 ± 0.060^c^	5.37 ± 0.028^b^	4.98 ± 0.110^a^	＜0.001	＜0.001	0.738
22:5n-3	0.15 ± 0.002^c^	0.10 ± 0.002^a^	0.13 ± 0.000^b^	＜0.001	＜0.001	＜0.001
22:6n-3	4.73 ± 0.099^b^	3.85 ± 0.011^a^	3.65 ± 0.009^a^	＜0.001	＜0.001	0.003
∑n-3 PUFA	11.05 ± 0.056^c^	9.58 ± 0.053^b^	9.07 ± 0.063^a^	＜0.001	＜0.001	0.001
∑LC-PUFA	11.26 ± 0.071^c^	9.71 ± 0.081^b^	9.39 ± 0.058^a^	＜0.001	＜0.001	＜0.001
n3/n6 PUFA	3.67 ± 0.153^c^	2.70 ± 0.070^b^	1.94 ± 0.045^a^	＜0.001	＜0.001	0.439
DHA/EPA	0.74 ± 0.015^b^	0.72 ± 0.002^ab^	0.69 ± 0.013^a^	0.039	0.014	0.882

FO = fish oil; CSO = cottonseed oil; ∑SFA = total saturated fatty acids; ∑MUFA = total mono-unsaturated fatty acids; ∑n-6PUFA = total n-6 polyunsaturated fatty acids; ∑n-3PUFA = total n-3 polyunsaturated fatty acids; ∑LC-PUFA = total long-chain polyunsaturated fatty acids; PUFA = polyunsaturated fatty acids; DHA/EPA = 22:6n-3/20:5n-3.

^a-c^ Means in the same row with different letter superscripts show significant differences (*P* < 0.05). Data was provided as mean ± SEM of three replications (*n* = 3).

**Fig. 3:**
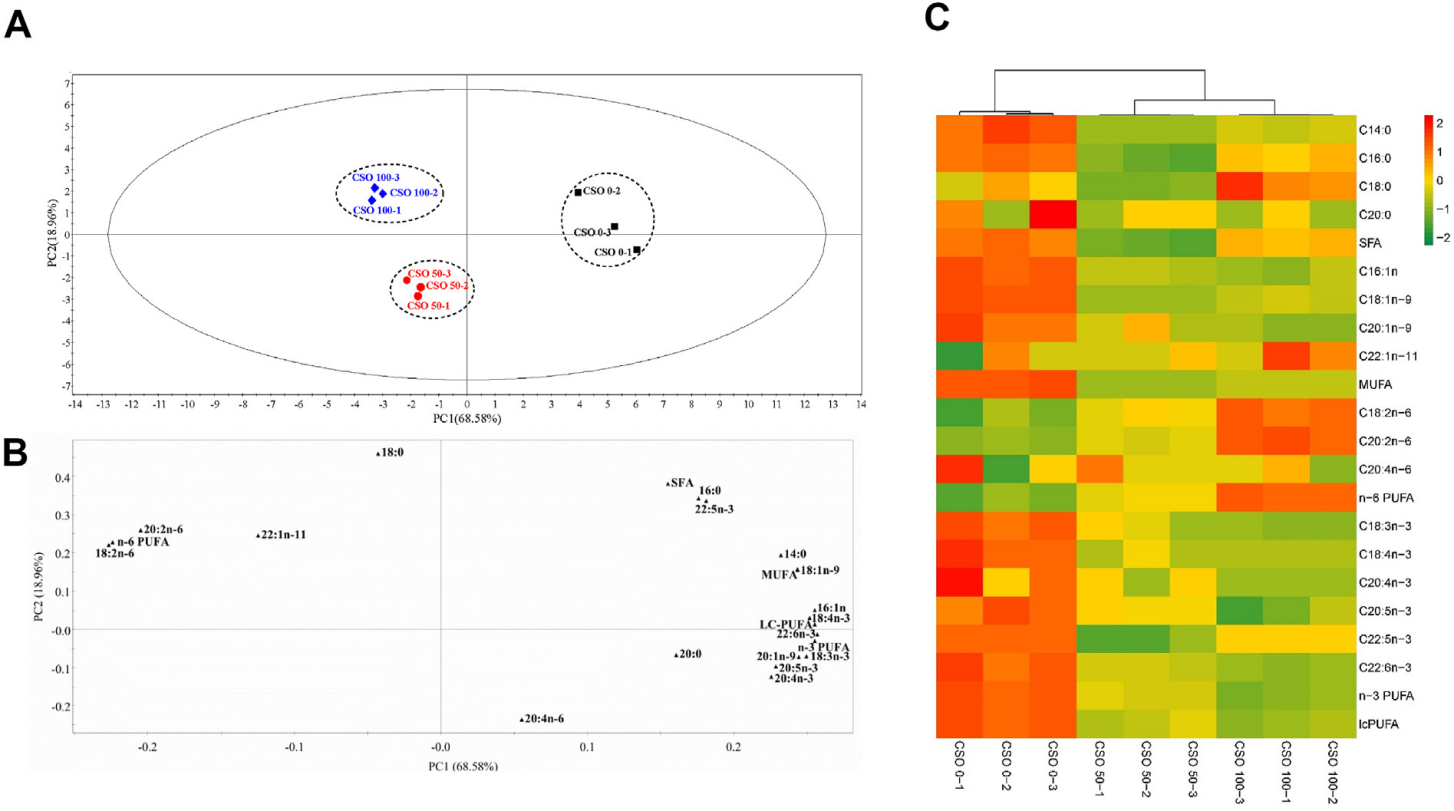
Principal component analysis (PCA) modeling of muscle fatty acids data (A and B); visualized heat map of muscle fatty acids data (C). CSO 0-1: substitution of fish oil with 0% cottonseed oil - 1; CSO 0-2: substitution of fish oil with 0% cottonseed oil - 2; CSO 0-3: substitution of fish oil with 0% cottonseed oil - 3; CSO 50-1: substitution of fish oil with 50% cottonseed oil - 1; CSO 50-2: substitution of fish oil with 50% cottonseed oil - 2; CSO 50-3: substitution of fish oil with 50% cottonseed oil - 3; CSO 100-1: substitution of fish oil with 100% cottonseed oil - 1; CSO 100-2: substitution of fish oil with 100% cottonseed oil - 2; CSO 100-3: substitution of fish oil with 100% cottonseed oil – 3. SFA = saturated fatty acids; MUFA = mono-unsaturated fatty acids; n-6PUFA = n-6 polyunsaturated fatty acids; n-3PUFA = n-3 polyunsaturated fatty acids; LC-PUFA = long-chain polyunsaturated fatty acids; DHA/EPA = 22:6n-3/20:5n-3.

As shown in Fig. 3A and B, according to the PCA score plot alongside the fatty acid compositions in the muscle, the first two PC collectively accounted for 87.54% of the total variation, with individual contributions of 68.58% and 18.96%, respectively (Fig. 3A). On the left side of PC1, both LA and ∑n-6 PUFA exhibited high correlations with the CSO-100 group. ALA and ∑LC-PUFA, on the other hand, were strongly associated with the CSO-0 group (Fig. 3B).

As shown in Fig. 3C, according to the heat maps for Pearson's correlation analysis in the muscle, fatty acid compositions showed high contents in the CSO-0 group. The contents of LA and ∑n-6PUFA were highest in the CSO-100 group.

#### 3.4.3 Composition of volatile substances in muscle

The effects of dietary substitution of FO with CSO on volatile compound in muscle of the swimming crabs were shown in Table 11. The results showed that there were 4 aldehydes, 5 esters, 1 hydrocarbon, 2 aromatic compounds and 8 miscellaneous compounds. The maximum aldehydes and esters were detected in the crabs fed with CSO-50 diet (*P* < 0.05). The total content of aromatics and miscellaneous compounds decreased with the increase of dietary replacement of FO with CSO (*P* < 0.05). The hydrocarbon dodecane was not detected in the CSO-0 group.

**Table 11:** Effects of dietary substitution of FO with CSO on volatile compound (ng/g) in the muscle for swimming crabs.

Item	Substitution of FO with CSO, %			*P-*value		
	0	50	100	ANOVA	Linear	Quadratic
**Aldehydes (4)**						
Nonanal	49.32 ± 14.721	37.58 ± 6.819	17.09 ± 1.690	0.125	0.052	0.717
Decanal	nd^a^	nd^a^	4.87 ± 1.778^b^	0.023	0.015	0.101
2,5-Dihydroxybenzaldehyde	nd^a^	18.22 ± 1.487^c^	6.62 ± 1.412^b^	＜0.001	0.008	＜0.001
Vanillin, TBDMS derivative	nd^a^	nd^a^	5.23 ± 1.231^b^	0.003	0.002	0.023
Total	49.32 ± 14.719	55.8 ± 5.340	33.81 ± 1.851	0.289	0.274	0.249
**Esters (5)**						
Tributyl phosphate	nd^a^	90.78 ± 4.312^b^	nd^a^	＜0.001	1.000	＜0.001
Oxime-, methoxy-phenyl-_	78.46 ± 9.304^b^	58.31 ± 2.261^ab^	32.66 ± 3.656^a^	0.007	0.002	0.692
Octanoic acid, ethyl ester	nd^a^	17.49 ± 5.001^ab^	22.2 ± 5.852^b^	0.028	0.012	0.285
2-(Benzyloxy) ethyl methyl carbonate	nd^a^	7.53 ± 1.064^b^	nd^a^	＜0.001	1.000	＜0.001
Carbonic acid, hexadecyl phenyl ester	nd^a^	8.16 ± 0.029^b^	5.75 ± 1.472^b^	0.001	0.003	0.002
Total	78.46 ± 9.291^a^	184.37 ± 7.050^b^	64.27 ± 8.194^a^	＜0.001	0.269	＜0.001
**Hydrocarbons (1)**						
Dodecane	nd^a^	8.96 ± 0.744^b^	12.74 ± 2.920^b^	0.005	0.002	0.269
**Aromatics (2)**						
Ethylbenzene	nd^a^	nd^a^	3.70 ± 1.024^b^	0.006	0.004	0.042
Benzene, 1,3-dimethyl-	19.99 ± 3.661^b^	10.98 ± 1.389^ab^	7.74 ± 2.669^a^	0.047	0.020	0.425
Total	19.99 ± 3.658	10.98 ± 1.391	11.44 ± 3.540	0.140	0.094	0.252
**Miscellaneous compounds (8)**						
Cyclotrisiloxane, hexamethyl-	315.38 ± 49.061^b^	285.50 ± 34.542^b^	106.41 ± 5.820^a^	0.011	0.005	0.131
Cyclotetrasiloxane, octamethyl-	78.13 ± 5.101^b^	nd^a^	nd^a^	＜0.001	＜0.001	＜0.001
Cyclopentasiloxane, decamethyl-	141.54 ± 66.318	111.08 ± 11.981	75.12 ± 9.122	0.526	0.277	0.956
Cyclohexasiloxane, dodecamethyl-	77.52 ± 38.040	48.89 ± 4.011	32.19 ± 2.277	0.399	0.196	0.886
Cycloheptasiloxane, tetradecamethyl-	58.91 ± 8.560	44.57 ± 7.265	37.59 ± 10.334	0.292	0.138	0.744
Dicyclopentadiene diepoxide	nd^a^	nd^a^	7.72 ± 1.030^b^	＜0.001	＜0.001	0.002
4-Ethylbenzamide	nd^a^	28.59 ± 5.024^b^	21.90 ± 3.643^b^	0.003	0.005	0.007
Heptasiloxane, 1,1,3,3,5,5,7,7,9,9,11,11,13,13-tetradecamethyl-	nd^a^	23.15 ± 1.032^c^	8.86 ± 1.781^b^	＜0.001	0.002	＜0.001
Total	648.14 ± 130.744^b^	513.17 ± 28.640^ab^	267.89 ± 15.552^a^	0.035	0.014	0.584

nd = volatile substance is not detected; FO = fish oil; CSO = cottonseed oil.

^a,b^ Means in the same row with different letter superscripts show significant differences (*P* < 0.05). Data was provided as mean ± SE of three replications (*n* = 3).

**Fig. 4:**
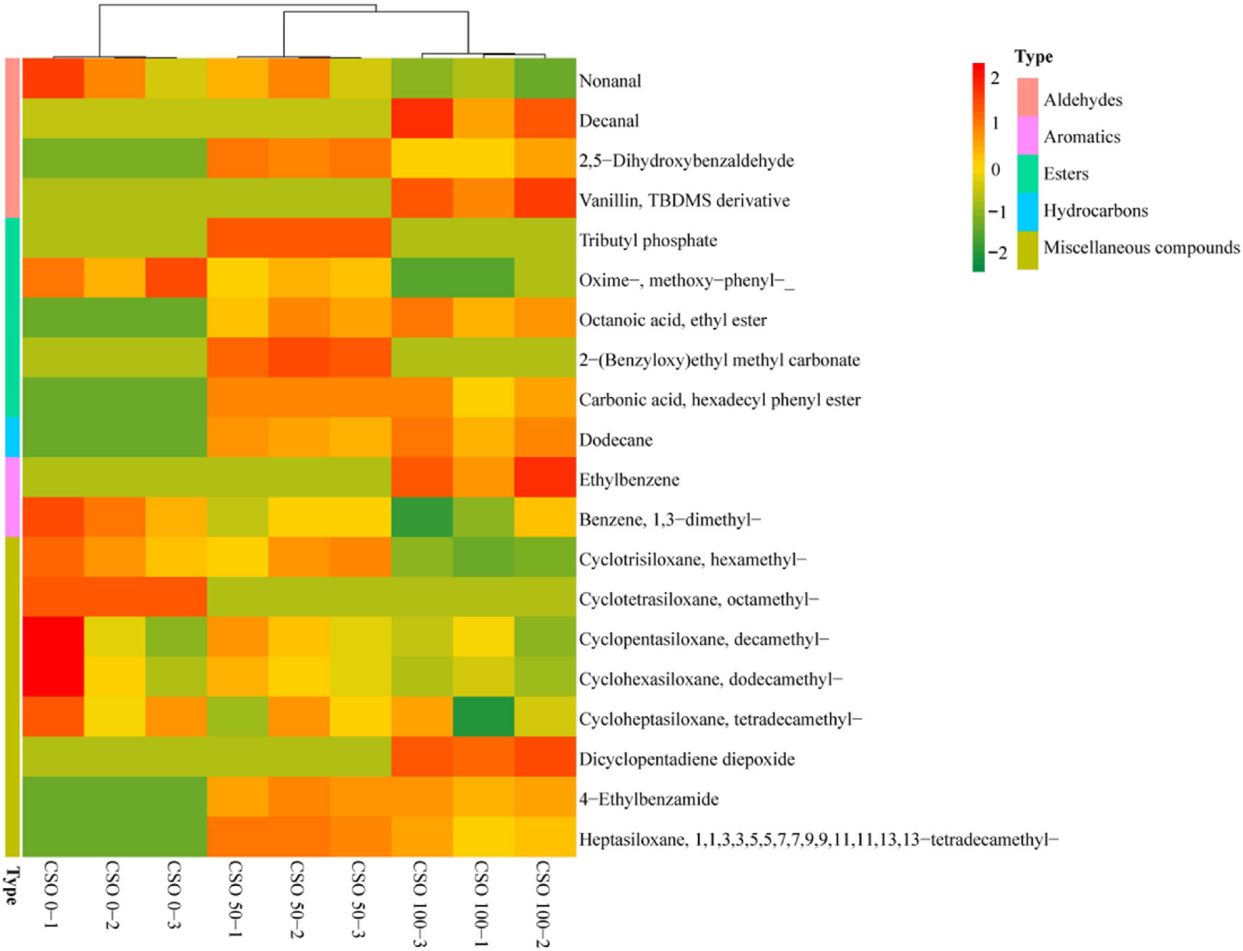
Heat map visualization of volatile compound data in the muscle of the swimming crabs (compound types are distinguished by the color block on the far left of heat map, peach for aldehydes, light pink for aromatics, green for esters, blue for hydrocarbons, and mustard yellow for others). CSO 0-1: substitution of fish oil with 0% cottonseed oil - 1; CSO 0-2: substitution of fish oil with 0% cottonseed oil - 2; CSO 0-3: substitution of fish oil with 0% cottonseed oil - 3; CSO 50-1: substitution of fish oil with 50% cottonseed oil - 1; CSO 50-2: substitution of fish oil with 50% cottonseed oil - 2; CSO 50-3: substitution of fish oil with 50% cottonseed oil - 3; CSO 100-1: substitution of fish oil with 100% cottonseed oil - 1; CSO 100-2 = substitution of fish oil with 100% cottonseed oil - 2; CSO 100-3: substitution of fish oil with 100% cottonseed oil – 3.

The effects of dietary substitution of FO with CSO on heat map visualization of volatile compound data in the muscle of the swimming crabs are presented in Fig. 4. Red indicates a high value and green indicates a low value. Two CSO supplementation groups (CS0-50 group and CSO-100 group) can be observed clustered together. The most volatile substances were occurred at crabs fed with CSO-50 and CSO-100 groups. Crabs fed with the CSO-0 diet showed higher concentrations of nonyl aldehyde, oxime, methoxyphenyl, and most miscellaneous compounds.

### 3.5 Correlation analysis of fatty acids in diet, hepatopancreas and muscle

**Fig. 5:**
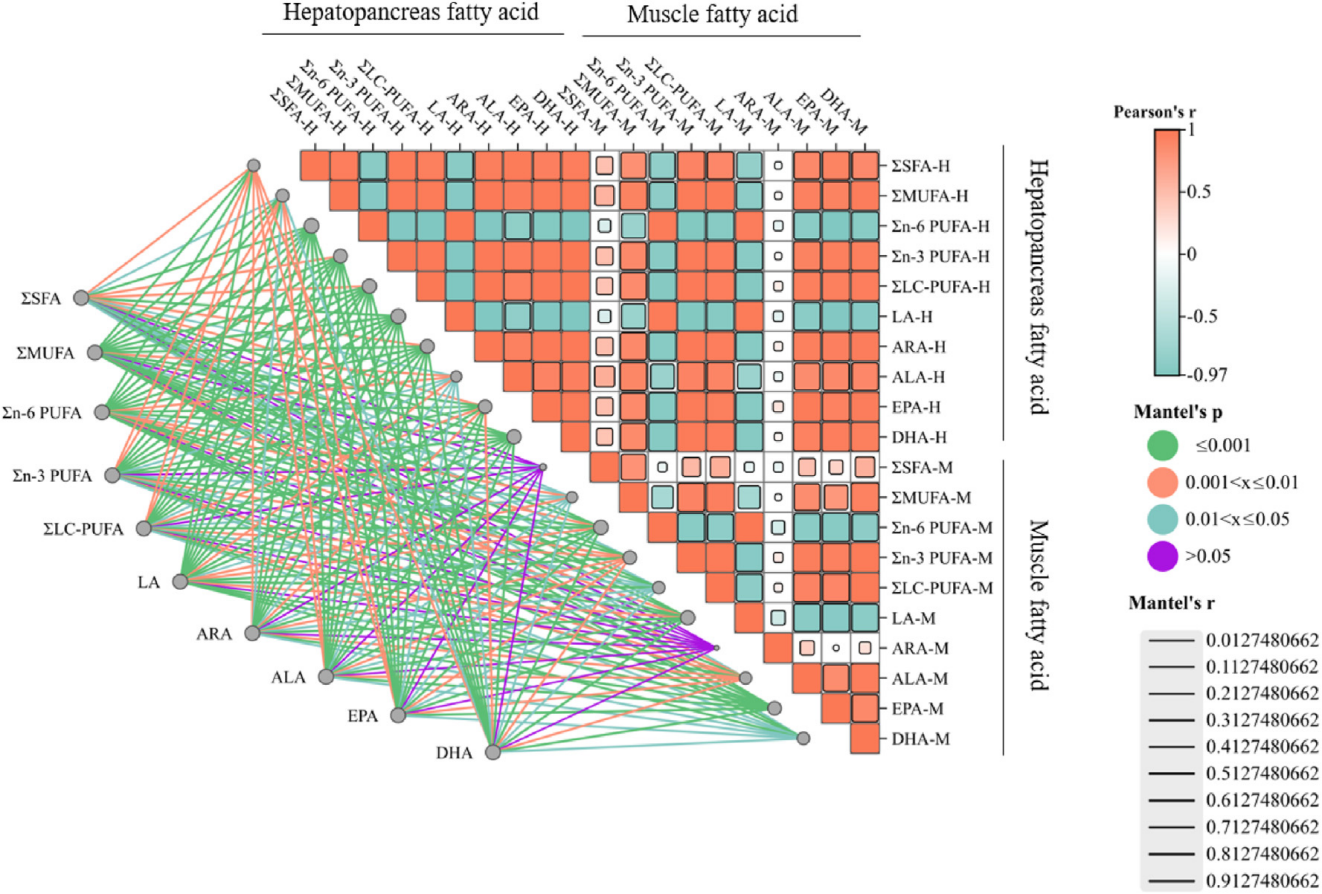
Correlations among dietary fatty acid composition, hepatopancreas fatty acid composition, and muscle fatty acid composition. The edge width of lines corresponds to Mantel's r for the statistics of related distance correlations; the color of lines denotes statistical significance. H = hepatopancreas; M = muscle; ∑SFA = total saturated fatty acids; ∑MUFA = total mono-unsaturated fatty acids; ∑n-6PUFA = total n-6 polyunsaturated fatty acids; ∑n-3PUFA = total n-3 polyunsaturated fatty acids; ∑LC-PUFA = total long-chain polyunsaturated fatty acids; DHA/EPA = 22:6n-3/20:5n-3; LA = linoleic acid; ARA = arachidonic acid; ALA = linolenic acid; DHA = docosahexaenoic acid; EPA = eicosapentaenoic acid.

As shown in Fig. 5, fatty acid composition in hepatopancreas and muscle is closely related to dietary fatty acid composition. The fatty acids profiles in diet (such as ∑SFA, ∑MUFA, ∑n-3 PUFA, ∑n-6 PUFA, ∑LC-PUFA, LA, ARA, ALA, EPA, and DHA) were significantly positively correlated with fatty acid composition in the hepatopancreas and partial muscle fatty acid (∑MUFA, ∑n-3 PUFA, ∑n-6 PUFA, ∑LC-PUFA, LA, ALA, EPA, and DHA) (*P* < 0.05). The contents of ∑SFA and ARA in muscle were not significantly associated with dietary fatty acid composition (*P* ＞ 0.05).

### 3.6 Expression of genes related to lipid and fatty acid metabolism in hepatopancreas

#### 3.6.1 Expression of genes related to lipid and fatty acid anabolism in hepatopancreas

**Fig. 6:**
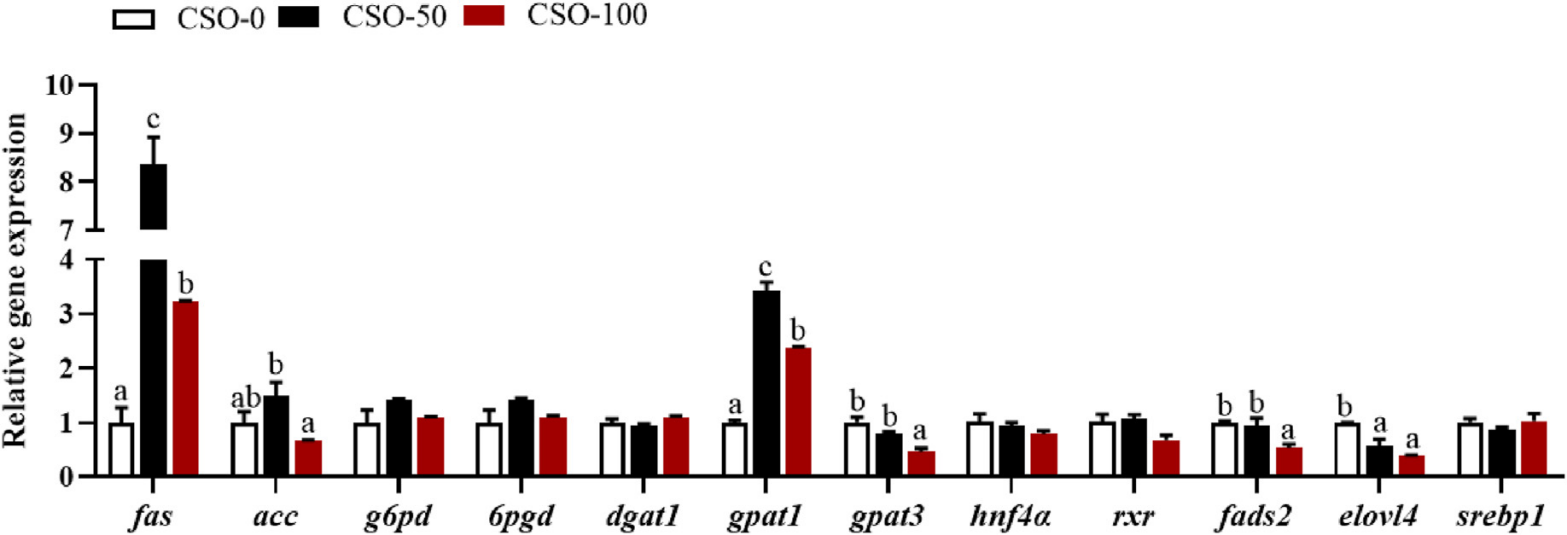
Effects of dietary substitution of fish oil (FO) with cottonseedoil (CSO) on the expression of lipid and fatty acid anabolism related genes in the hepatopancreas of swimming crabs. CSO-0: substitution of fish oil with 0% cottonseed oil; CSO-50: substitution of fish oil with 50% cottonseed oil; CSO-100: substitution of fish oil with 100% cottonseed oil. *fas* = fatty acid synthase; *acc* = acetyl-CoA carboxylase; *g6pd* = glucose 6-phosphate dehydrogenase; *6pgd* = 6-phosphogluconate dehydrogenase; *dgat1 =* diacylglycerol acyltransferase 1; *gpat1 =* glycerol-3-phosphate acyltransferase 1; *gpat3 =* glycerol-3-phosphate acyltransferase 3; *hnf4α* = hepatocyte nuclear factor 4-alpha; *rxr* = retinoid X receptor; *fads2* = fatty acyl desaturase 2; *elovl4* = elongase 4; *srebp1* = sterol regulatory element-binding protein 1. ^a-c^ Means values of bars for the same parameter with different superscript letters are significantly different (*P* < 0.05). Data was provided as mean ± SE of three replications (*n* = 3).

As shown in Fig. 6, in terms of lipid anabolic metabolism, the relative expression levels of fatty acid synthetase (*fas*), acetyl-CoA carboxylase (*acc*) and glycerol-3-phosphate acyltransferase 1 (*gpat1*) in hepatopancreas of swimming crabs fed with the CSO-50 diet were significantly higher than those fed the other diets (*P* < 0.05). The relative expression levels of gluconate 6-phosphate dehydrogenase (*g6pd*) and glucose 6-phosphate dehydrogenase (*6pgd*) were not significantly affected by dietary substitution of FO with CSO (*P* > 0.05). For fatty acid anabolism, the relative expression levels of fatty acyl desaturase 2 (*fads2*) and elongase 4 (*elovl4*) down-regulated with the increased CSO substitution (*P* < 0.05). However, there were no significant differences in hepatocyte nuclear factor 4-alpha (*hnf4α*), retinoid X receptor (*rxr*), and sterol regulatory element-binding protein 1 (*srebp1*) expression among all treatments (*P* > 0.05).

#### 3.6.2 Expression of genes related to fatty acid transport and catabolism in hepatopancreas

**Fig. 7:**
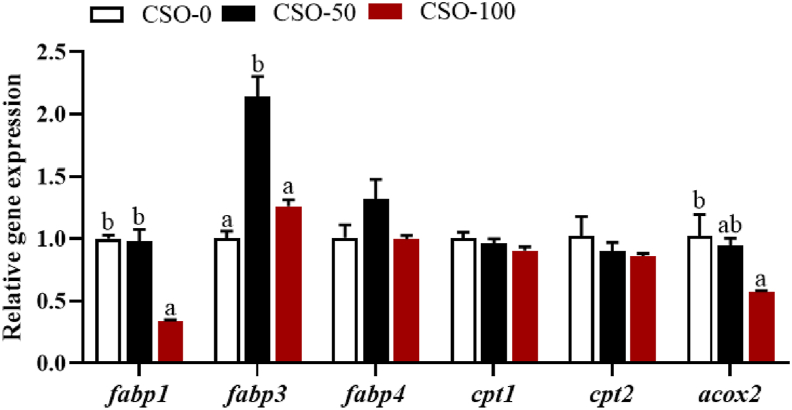
Effects of dietary substitution of fish oil (FO) with cottonseedoil (CSO) on the expression of fatty acid transport and catabolism related genes in the hepatopancreas of the swimming crabs. CSO-0: substitution of fish oil with 0% cottonseed oil; CSO-50: substitution of fish oil with 50% cottonseed oil; CSO-100: substitution of fish oil with 100% cottonseed oil. *fabp1* = fatty acid binding protein 1; *fatp3* = fatty acid transport proteins 3; *fatp4* = fatty acid transport proteins 4; *cpt1* = carnitine palmitoyltransferase 1; *cpt3* = carnitine palmitoyltransferase 3; *acox2* = acyl-CoA oxidase 2. ^a,b^ Means values of bars for the same parameter with different superscript letters are significantly different (*P* < 0.05). Data was provided as mean ± SE of three replications (*n* = 3).

As shown in Fig. 7, for fatty acid transport, the relative expression level of fatty acid binding protein 3 (*fabp3*) in the hepatopancreas of swimming crabs in the CSO-50 group was significantly higher than those fed the other diets (*P* = 0.001). The relative expression level of fatty acid binding protein 1 (*fabp1*) in the hepatopancreas down-regulated considerably when FO was totally replaced with CSO (*P* < 0.001). However, there was no significant difference in the relative expression level of fatty acid transporter protein 4 (*fatp4*) among all treatments. For fatty acid catabolism, the relative expression levels of carnitine palmitoyltransferase 1 (*cpt1*) and carnitine palmitoyltransferase 2 (*cpt2*) in hepatopancreas of the swimming crabs were not significantly affected by substitution of FO with CSO (*P* > 0.05). The relative expression level of acyl-CoA oxidase 2 (*acox2*) in hepatopancreas down-regulated with the increase of dietary substitution of FO with CSO (*P* = 0.045).

## 4 Discussion

The results of present study demonstrated that it is feasible to replace FO with CSO in feed, and 50% substitution of FO with CSO has no adverse effects on growth performance and survival, while completely replacement of FO in feed with CSO can significantly reduce the growth performance and survival rate of swimming crabs. These results are similar to previous studies on rainbow trout (Güler and Yildiz, 2011), black seabream (*Acanthopagrus schlegelii*) (Wu et al., 2022), European sea bass (*Dicentrarchus labrax*) (Eroldoğanet al., 2012), juvenile red drum (*Sciaenops ocellatus* L.) and hybrid striped bass (*Morone chrysops* × *M. saxatilis* S.) (Candelaria et al., 2022). Prior research also indicated that replacing 50% or even 100% of FO with other vegetable oils (canola, palm, linseed, and soybean oil) did not negatively affect the growth performance of swimming crabs (Long et al., 2019; Lu et al., 2020; Yang et al., 2023). Conversely, high levels of CSO substitution for FO can decrease growth performance in Nile tilapia (*Oreochromis Niloticus*) (Li et al., 2016), clamworm (*Perinereis aibuhitensis*) (Lu et al., 2016), and gilthead seabream (Wassef et al., 2015). The decreased growth performance may be associated with high CSO content affecting feed palatability (Wang et al., 2020a). However, this effect was not observed in the present study, possibly because the diet was based on fish meal and plant protein. When FO was replaced by CSO, a diet with appropriate levels of DHA + EPA maintained normal growth in swimming crabs (Wassef et al., 2015; Wang et al., 2021). Moreover, it also may be due to the different ability to utilize dietary fatty acids for different aquatic animals (Aksakal et al., 2023). Meanwhile, the stability of diets supplemented with CSO may be improved due to its richness in n-6 fatty acids and antioxidant tocopherols (Wassef et al., 2015). It may also be due to the beneficial effects of vitamin E-rich CSO on the growth of crustaceans and the fact that crustaceans generally require lower levels of vitamin E than fish (He and Lawrence, 1993; Efrizal et al., 2018; El-Sayed and Izquierdo, 2022). Therefore, compared with fish, CSO can replace 50% of FO without causing a significant reduction in growth performance and survival of swimming crabs.

The results of present study showed that the lipid content in the hepatopancreas of swimming crabs fed with the CSO-50 and CSO-100 diets was significantly higher than that fed with the CSO-0 diet. Previous results suggested that high levels of CSO replacement led to lipid deposition in the liver of rainbow trout (Güler and Yildiz, 2011) and black seabream (Wu et al., 2022). Similar results were also reported for gilthead seabream (Wassef et al., 2015) and rainbow trout (Güler and Yildiz, 2011). Possible explanations for this result included an imbalance in the proportion of n-3/n-6 PUFA in the diet and the accumulation of lipids in hepatopancreas due to the high proportion of linoleic acid in the diet of CSO-50 and CSO-100 (Sargent et al., 2002). At the same time, in this study, NEFA and GLU contents in hemolymph increased significantly with increasing levels of CSO substituted for FO. This result is consistent with previous studies where 80% CSO replacement of FO significantly increased NEFA content in black seabream serum (Wu et al., 2022). Excessive accumulation of lipid metabolites in the blood is always associated with impaired or disturbed lipolytic metabolic function (Du et al., 2013; Lei et al., 2016). This suggests that lipid metabolism in swimming crabs may be damaged when CSO completely substitutes FO, hindering normal glucose transport in the hemolymph and resulting in increased glucose levels. Similar results were reported for Chinese mitten crab (*Eriocheir sinensis*) (Ma, 2018). It has also been reported that total substitution of dietary FO with soybean oil induces insulin dysfunction, raising blood GLU content in large yellow croaker (*Larimichthys crocea*) (Gu et al., 2019), consistent with the present study's results. The observation of histological structure revealed that the number of B cells significantly increased when dietary CSO substitution for FO increased from 0% to 100%. However, in this study, ALT and AST activities in the hemolymph did not increase with higher CSO replacement levels, and no signs of hepatopancreas injury were observed. These observations may simply be a physiological response of the liver adapting to dietary excess of linoleic acid, suggesting that these signs might be reversible (Alexis, 1997; Diaz-Lopez et al., 2009; Turchini et al., 2009). At the same time, it was speculated that the observations may also be due to the accumulation of more lipids in the CSO-50 group, which provides the necessary energy for the growth process - the growth performance of swimming crabs in the CSO-50 group being better than that in the CSO-100 group. Further research is needed to determine the impact of substituting 100% FO with CSO on glucose metabolism and hepatopancreatic health in swimming crabs.

In crustaceans, lipids are considered to be the main organic reserve substances that control various physiological metabolic processes (O'Connor and Gilbert, 1968). Generally speaking, lipids need to be transported to various tissues via serum as a carrier, and the hepatopancreas is a crucial organ for lipid metabolism (Yuan et al., 2019; Wang et al., 2020b). Because of the difference in fatty acid composition between FO and CSO, replacing FO with CSO may affect the lipid metabolism of swimming crabs (Liu et al., 2016; Zhao et al., 2016). In the present study, the contents of TAG and T-CHO in hemolymph and the content of T-CHO in the hepatopancreas were significantly higher in crabs fed with CSO-50 than those fed with the CSO-0 and CSO-100 diets. Similar results reported that when the level of FO that was replaced by mixed vegetable oils (soybean and rapeseed oil) exceeded 50%, it could significantly increase the hepatopancreatic TAG and T-CHO contents of swimming crabs (Long et al., 2019). The results suggested that the dietary substitution of FO with CSO was beneficial for the accumulation of TAG and the absorption and utilization of T-CHO in swimming crabs (Wu et al., 2019).

Changes in the composition of fatty acids from dietary lipid sources can affect the composition of volatile substances in the muscles of aquatic animals, as demonstrated in species such as swimming crabs (Yuan et al., 2020), Chinese mitten crab (Cong et al., 2020), gilthead seabream (Alexi et al., 2017) and yellow large croaker (*L. crocea*) (Mu et al., 2021). Aldehydes are the principal volatile components contributing to the flavor of crab flesh, due to their large amounts and low odor thresholds (Wang et al., 2016a; Wu et al., 2019). Nonanal and sunflower aldehyde were found to be obtained from OA oxidation, with nonanal specifically contributing to the grassy flavor notes in crab meat (Wang et al., 2016b). Esters are usually produced by esterification of acids and alcohols, Ethyl octanoate has a fruity-sweet flavor that masks fishy flavors and is a typical volatile substance in wine (Bell and Henschke, 2010; Cong et al., 2020). The present study indicated that 16 volatile substances detected in the muscle of swimming crabs were significantly affected by substituting FO with CSO, with six esters being notably influenced. The results showed that the content of nonanal in muscle slightly decreased with increasing levels of CSO substitution. Similar results were found for the FO group with significantly higher nonanal content than vegetable oil group (Yuan et al., 2020). Conversely, ethyl octanoate content was higher and undetectable in the control group. Therefore, we speculate that dietary replacement of FO with CSO may cause the muscles of swimming crabs to become less grassy and increase their sweetness. Additionally, heat map analysis showed that the composition of muscle volatile substances was similar to the fatty acid composition of muscle. The CSO-50 and CSO-100 groups clustered into one cluster with a relatively similar composition. This reaffirms that changes in fatty acid composition influence changes in volatile substances composition. As a lipid source, the primary difference between CSO and FO is in the fatty acid composition. Long-term intake of feeds with different fatty acid compositions further maps the fatty acid composition of aquatic animals (Bell et al., 2002; Turchini et al., 2011). According to the present study, increasing the CSO substitution level resulted in a significant upward trend in LA and ∑n-6 PUFA contents in hepatopancreas and muscle, ALA, ∑n-3 PUFA, and ∑LC-PUFA contents showed an opposite trend. These findings are consistent with the results of using soybean oil and rapeseed oil to replace FO in swimming crabs (Long et al., 2019). The results were also similar to those reported on some fish species (Güler and Yildiz, 2011; Eroldoğan et al.; Wassef et al., 2015; Wu et al., 2022). Combining heat maps and PCA analysis, the fatty acid composition data of the hepatopancreas and muscle were divided into 3 clusters corresponding to the 3 diet groups in this trial, with the CSO-50 and CSO-100 groups clustered together.

In lipid synthesis, fas and acc are key lipid-producing and rate-limiting enzymes in fatty acid synthesis (Qian et al., 2015). g6pd and 6pgd are key regulatory enzymes involved in providing the reduced coenzyme II (NADPH) that are key regulatory enzymes for fatty acid biosynthesis (Chen et al., 2012), and the initiation reaction of TAG synthesis is regulated by gpat as the rate-limiting enzyme (Yen et al., 2008; Castro et al., 2016). In the present study, the expression of *fas*, *acc*, and *gpat* in the hepatopancreas was highest in crabs fed with the CSO-50 diet. However, the expression of *6pgd* and *g6pd* was not significantly affected by replacing FO with CSO. Similar observations were also reported for Chinese mitten crab (*E. sinensis*) (Liu et al., 2016). This may be due to the increased MUFA content in the feed, which promotes the expression of genes related to fatty acid and TAG synthesis in the hepatopancreas (Ribeiro et al., 2008).

In the present study, CSO substitution for FO significantly down-regulated the expression of *fads2* and *elovl4*, key genes in the LC-PUFA synthesis pathway (Ting et al., 2020). In contrast, using n-6 PUFA and n-3 PUFA lipid sources to replace FO up-regulated the expression of these genes in crustaceans (Alhoshy et al., 2022). This may be caused by differing lipid sources and replacement levels. The expression of *fads2* and *elovl4* were found to be up-regulated when levels of dietary substitution of FO with CSO ranged from 20% to 40% but were found to be down-regulated with substitution levels above 40% (Wu et al., 2022). This result is consistent with the results of this study, where high levels (≥50%) of CSO replacement FO down-regulated the expression of *fads2* and *elovl4* genes. Although high levels of CSO inhibited fatty acid synthesis, there was no significant difference in *fabp1* expression between the CSO-50 and CSO-0 groups during fatty acid transport. However, *fabp3* expression in the CSO-50 group was significantly higher than in the CSO-0 group, indicating that 50% CSO replacement promoted fatty acid transport to some extent in swimming crabs. Further research is needed to investigate the effects of different levels of CSO substitution on fatty acid transport in swimming crabs. Fatty acid catabolism is implemented mainly by β-Oxidation as well as the CPT family plays a key role in controlling and regulating fatty acid β-Oxidation (McGarry and Brown, 1997). In the present study, there was no significant difference in the expression of *cpt1* and *cpt2* among different dietary groups as the CSO replacement level increased, although a decreasing trend was observed. Similarly, when soybean oil and rapeseed oil replaced FO in Chinese mitten crab diets, the expression of *cpt1α* and *cpt2* in the hepatopancreas first increased and then decreased with higher dietary substitution levels. This may be due to the excessive LA content in the diets (Liu et al., 2018). Therefore, it was also speculated that the substitution of CSO led to an increase in dietary LA content, which down-regulated genes related to fatty acid catabolism.

## 5 Conclusion

In summary, the substitution of 50% FO with CSO had no adverse effects on the growth performance and feed utilization of swimming crabs. However, the complete substitution of FO with CSO in the diet significantly reduced the growth performance and survival of the crabs. Replacing 50% FO with CSO increased the lipid content of hepatopancreas, up-regulated the expression of lipid synthesis-related genes such as *fas*, *acc* and *gpat*, and effectively increased lipid accumulation in hepatopancreas. As the level of CSO substitution increased, the expression of *fads2* and *elovl4* genes associated with fatty acid anabolism in hepatopancreas were down-regulated. The contents of LA and ∑n-6 PUFA in the hepatopancreas and muscle showed an increasing trend with increasing substitution while the contents of ∑n-3 PUFA and ∑LC-PUFA showed the opposite trend. Additionally, replacement of FO with CSO influenced the concentration of volatile substances in the muscle, thus affecting the muscle flavor of swimming crabs.

## CRediT author statement

**Tiantian Xu:** Writing – Original draft, Data curation, Conceptualization. **Zheng Yang:** Validation, Data curation. **Shichao Xie:** Software, Formal analysis. **Tingting Zhu:** Project administration, Investigation. **Wenli Zhao:** Data curation. **Min Jin:** Funding acquisition, Formal analysis. **Qicun Zhou:** Writing – Review & Editing, Supervision.

## Declaration of competing interest

We declare that we have no financial and personal relationships with other people or organizations that can inappropriately influence our work, and there is no professional or other personal interest of any nature or kind in any product, service and/or company that could be construed as influencing the content of this paper.
